# Influence of vehicle expertise on acceleration profile preferences in electric vehicles

**DOI:** 10.1371/journal.pone.0325331

**Published:** 2025-06-04

**Authors:** Seonghyeon Kim, Jaesik Yang, Eunju Jeong

**Affiliations:** 1 Chair of Acoustics and Haptics, Institute of Acoustics and Speech Communication, Faculty of Electrical and Computer Engineering, Technische Universität Dresden, Dresden, Germany; 2 Hyundai Motor Company, Hwaseong-si, Gyeonggi-do, South Korea; 3 Department of Music Therapy, Graduate School, Ewha Womans University, Seodaemun-gu, Seoul, South Korea; Yalova University, TÜRKIYE

## Abstract

This study examines how professional expertise influences acceleration profile preferences by comparing evaluations from development experts in internal combustion engine vehicles (ICEVs) and electric vehicles (EVs). Subjective evaluations were conducted under light and middle tip-in acceleration conditions, where participants assessed five distinct acceleration profiles defined by maximum jerk, acceleration gradient, and jerk kurtosis. Results indicated that ICEV experts preferred profiles emphasizing stability and smooth transitions, while EV experts showed more balanced preferences for responsiveness and smoothness. Under light tip-in acceleration, ICEV experts demonstrated strong negative correlations between subjective preference and maximum jerk (*r* = −0.85) and jerk kurtosis (*r* = −0.75), indicating aversion to sharp transient dynamics. EV experts showed weaker correlations (*r* = −0.26 and *r* = −0.63, respectively), suggesting more flexible perception of these characteristics. Under middle tip-in acceleration, EV experts displayed strong negative correlations with maximum jerk (*r* = −0.82) and jerk kurtosis (*r* = −0.89), while ICEV experts exhibited negligible or weak associations with these parameters. These findings demonstrate that professional background significantly influences acceleration profile preferences, with ICEV experts valuing traditional driving dynamics and EV experts accepting more responsive characteristics typical of electric drivetrains. The results offer practical guidelines to improve user satisfaction and facilitate a smoother transition from ICEVs to EVs by aligning vehicle drivability characteristics with different user expectations based on their professional expertise and driving experience.

## 1. Introduction

Electric vehicles (EVs) have emerged as a sustainable alternative to conventional internal combustion engine vehicles (ICEVs), offering substantial potential to reduce environmental pollution, fossil fuel dependency, and greenhouse gas emissions [1–5]. Reflecting the accelerating global shift toward sustainable mobility, EV adoption has increased markedly over the past year. According to the Global EV Outlook 2024 by the International Energy Agency (IEA) [6], global sales of electric cars exceeded 14 million units in 2023, which corresponds to approximately 18 percent of total car sales. This represents a 35 percent increase compared to 2022. As a result, the total number of electric cars in operation worldwide reached more than 40 million by the end of 2023. This growth is notably concentrated in a few major markets. China, the European Union, and the United States together accounted for over 95 percent of global EV sales. China alone accounted for over 60% of global EV sales in 2023, reinforcing its dominant role in the market. Meanwhile, EVs accounted for approximately 33 percent of new car sales in China and about 21 percent in the European Union. Norway continued to lead globally in terms of market share, with nearly 95 percent of new car registrations being electric. Although these figures highlight strong momentum in select regions, the global adoption of EVs remains unevenly distributed. In many countries, market penetration remains limited due to infrastructure shortcomings, economic barriers, and limited public awareness [7,8].

To better understand what drives user adoption and satisfaction with EVs despite these structural challenges, it is important to consider their core technical and experiential advantages over ICEVs. Compared to ICEVs, EVs provide significant advantages, such as lower maintenance costs and improved drivability [9–11], both of which have received positive user feedback. Specifically, EVs deliver high torque instantaneously during acceleration, enabling smoother and more precise driving experiences than ICEVs [12,13]. Previous studies indicate that the simplified powertrain of EVs reduces vibrations and jerks within the driveline, contributing to an enhanced driving quality that is challenging to achieve with ICEVs [14,15]. Furthermore, EVs equipped with electric motors demonstrate robust and stable acceleration performance even at high speeds, offering drivability superior to that of ICEVs [16]. Recent studies further support these technical advantages by introducing advanced motion planning and vehicle dynamics modeling techniques. For instance, lateral dynamics-inspired neural network models and dual-attention-based AGRU controllers have shown promise in improving EV control precision under limited data conditions [17–19]. In parallel with these advancements in EV-specific technologies, recent studies in intelligent transportation systems, vehicle control architectures, and networked driving environments have laid a solid technological foundation for the advancement of future mobility research. A wide range of research has investigated emerging methods in motion control [17,20], data-driven personalization [21,22], vehicular networking [23–25], and sustainable transportation systems [26–28], reflecting the multifaceted innovations that support the evolution of eco-friendly vehicles.

The technical advantages of EVs are further highlighted by user experience, with research indicating significant differences in preferences and attitudes between individuals who have experienced EVs and those who have not. Jensen et al. found that users’ preferences shifted substantially after three months of EV use [29]. Initial concerns regarding driving range and battery longevity diminished after use, while positive evaluations of fuel efficiency and driving performance increased. Moreover, the difference in preferences between experienced and inexperienced users becomes more pronounced. Those unfamiliar with EVs often focus on limitations such as driving range and charging infrastructure, whereas experienced users place greater value on smooth acceleration, low noise, and instant torque [30]. These findings emphasize the role of direct EV experience in lowering psychological barriers and fostering a preference for EVs over ICEVs.

Despite their environmental benefits and energy efficiency, EVs are sometimes perceived as underperforming compared to ICEVs in terms of driving performance [16,31,32]. Skippon reported that while EV users appreciated smooth and precise acceleration at lower speeds, they noted insufficient dynamic performance at higher speeds [32]. Similarly, Poornesh et al. observed that EVs perform efficiently in low-speed driving conditions but exhibit limitations in acceleration and top speed during high-speed scenarios, resulting in reduced satisfaction compared with ICEVs [16]. Such disparities in driving performance are not solely technological but also affect drivers’ mental models [33,34]. The differences in driving characteristics between EVs and ICEVs may influence user preferences and highlight the need for further research on how such mental models shape perceptions of EV drivability.

In previous work, Kim and Yang explored EV acceleration preferences using subjective evaluations from 15 participants [35]. While the findings provided valuable insights into general preferences for acceleration profiles, the study did not examine the impact of familiarity with EVs on evaluation outcomes. This represents a notable limitation, as individuals familiar with EVs and those accustomed to ICEVs are likely to have different expectations and mental models influenced by their prior driving experiences. These differences may play a significant role in shaping preferences for acceleration profiles. In addition to user experience and psychological familiarity, recent international studies emphasize that driver behavior plays a pivotal role in determining the environmental performance of electric vehicles. For instance, Kubik et al. conducted a comparative analysis of internal combustion and electric vehicles within an operational car-sharing context and found that electric vehicles resulted in carbon dioxide emission reductions ranging from 14 percent to 65 percent [36]. The magnitude of these reductions varied according to contextual factors including trip length, route characteristics, ambient temperature, and, most notably, driving behavior. The study also indicated that in specific scenarios involving short-distance, high-speed urban trips conducted under low-temperature conditions and with inefficient driving, electric vehicles could exhibit higher emissions than internal combustion engine vehicles due to increased energy demand. These results emphasize that the environmental benefits of electric vehicles are not guaranteed by technology alone but are critically dependent on driver engagement with energy-efficient practices. Other empirical research supports this finding, demonstrating that driver acceleration patterns are strongly associated with variations in energy consumption and emissions under real-world driving conditions [37]. Furthermore, scenario-based modeling has projected that large-scale adoption of energy-conscious driving behaviors could prevent over 400 million tons of carbon dioxide emissions by the year 2050 [38]. Considering these findings collectively, it can be concluded that driver behavior should be regarded as a critical systemic factor in the design and implementation of environmentally effective electric vehicle technologies. In parallel, Chu et al. proposed an optimization framework that minimizes battery capacity loss during acceleration by controlling the velocity trajectory of intelligent electric vehicles [39]. Their model integrates battery aging behavior into the design of acceleration profiles, demonstrating that optimized trajectories can simultaneously reduce energy consumption and prolong battery life without compromising acceleration performance. These studies support the importance of aligning technical profile design with driver behavior and real-world operating conditions.

In this context, the study by Egbue and Long provides further insights by highlighting several barriers to EV adoption [40]. It emphasizes the significant influence of socio-technical factors on consumer attitudes, including the role of mental models and prior experiences. According to their findings, technological enthusiasts and early adopters are more inclined to accept EVs when they perceive them to offer superior performance and reliability. In contrast, traditional consumer groups may exhibit resistance to transitioning to EVs due to established habits and perceived risks associated with adopting new technologies. These observations highlight the importance of addressing familiarity and mental models to better understand and accommodate various user preferences in EV design and adoption strategies. Moreover, Helmbrecht et al. reported that drivers exhibited aggressive acceleration and deceleration patterns during initial EV use but gradually adopted smoother and more stable driving behaviors after five months of experience [41]. This indicates that users require a learning period to adapt to EV-specific driving dynamics, such as high torque and unique acceleration characteristics. Additionally, Wang et al. further investigated eco-driving behaviors and found that EV users exhibit calmer driving patterns and a greater willingness to trade travel time for energy efficiency compared to ICEV users [42]. This reflects how EV-specific driving dynamics influence user habits and mental models. Jensen et al. analyzed data from 107 households that drove both EVs and ICEVs, revealing that EV drivers exhibited higher sensitivity to travel time and distance when choosing routes compared to ICEV drivers. Furthermore, route directness was evaluated differently depending on the vehicle type, reflecting differences in the mental models of EV and ICEV drivers [43]. Moreover, Tiwary et al. demonstrated that energy consumption in EVs is significantly influenced by drivers’ acceleration and deceleration patterns, with substantial variability observed even under identical distances and environmental conditions [44]. The study emphasized the need for EV drivers to adapt to eco-driving practices more effectively than ICEV drivers, highlighting new demands placed on their mental models. These findings highlight the importance of accounting for mental model differences when evaluating acceleration preferences between experienced and inexperienced EV users. Failure to account for these differences may constrain the interpretation of preference data and impede efforts to optimize EV design. Therefore, it is essential to conduct further studies that stratify participants according to their familiarity with EVs to investigate how such differences affect preference formation and subjective evaluations. Table 1 provides an overview of representative prior studies related to EV user experience, driving behavior, and acceleration preference. Although prior research has addressed EV drivability, energy efficiency, and behavioral adaptation, few studies have compared subjective acceleration preferences as influenced by technical expertise. The present study aims to address this research gap through a controlled comparative experiment involving EV and ICEV development experts.

**Table 1:** Summary of related studies on EV driving experience and acceleration preference.

Study	Main focus	Methodology	Participants	EV familiarity considered	Key limitations	Relevance to acceleration preference
**Jensen et al.** [30]	Long-term changes in EV user perceptions	Longitudinal user study	107 households	Yes	Did not examine driving attributes	Showed perception evolution post-EV use
**Skippon** [32]	Subjective performance comparison of EVs vs. ICEVs	Questionnaire survey	General drivers	No	Subjective only, no performance link	Reported EVs underperforming at high speeds
**Poornesh et al.** [16]	EV performance at different speeds	Controlled driving tests	Not specified	No	No user preference data	Identified performance gaps in speed domains
**Egbue & Long** [40]	Socio-technical barriers to EV adoption	Consumer survey	General public	Yes	No driving behavior analysis	Highlighted influence of mental models
**Kim & Yang** [35]	Evaluation of acceleration profiles in EVs	Subjective real-driving tests	15 drivers	No	No user type differentiation	Developed acceleration profiles
**Helmbrecht et al.** [41]	Behavioral adaptation in EV users	Field study (5 months)	EV users	Yes	No preference assessment	Showed adaptation over time
**Wang et al.** [42]	Comparison of eco-driving in EVs and ICEVs	Driving behavior monitoring	Matched EV and ICEV groups	Yes	Focused on efficiency, not feel	Reported calmer EV driving styles
**Tiwary et al.** [44]	Influence of acceleration on energy use	Energy consumption analysis	EV drivers	Yes	No preference analysis	Linked acceleration to energy use
**Chu et al.** [39]	Optimization of acceleration profiles	Simulation with battery modeling	N/A	No	No human factors considered	Proposed optimal trajectories
**Current study**	Effect of technical expertise on acceleration preferences	Real driving, paired comparison	15 EV & ICEV developers	Yes	Small expert sample	Compared subjective preferences by expertise

To address this gap in the literature, this study focuses on differences in EV acceleration preferences depending on technical familiarity and background. While prior research has explored general user perceptions and technical optimization, few studies have examined how professional experience and system familiarity influence subjective evaluations of acceleration profiles under real-world driving conditions. To contribute to this area, we conduct a comparative study using the tip-in acceleration profiles developed by Kim and Yang [35], which were originally evaluated based on subjective preferences from experienced drivers. Building upon previous research, this study distinguishes between EV and ICEV development experts among the 15 evaluators to examine how technical background influences preferences for key performance characteristics. Specifically, the study compares the acceleration profile preferences of these two groups, analyzing how system familiarity and underlying mental models influence their evaluations. Based on subjective assessments, it identifies preferred profile characteristics for each group, particularly in terms of initial acceleration response and shock mitigation. This study addresses two primary research questions: (1) Do differences in expertise lead to variations in preferences for acceleration profiles? (2) What dynamic characteristics are associated with these preferences in subjective evaluations? By addressing these questions, this study aims to identify the factors driving preference differences between the two expert groups and derive design guidelines for optimizing electric vehicle acceleration profiles. These guidelines aim to enhance user satisfaction and support a smoother transition from internal combustion engine vehicles to electric vehicles by addressing diverse user preferences.

## 2. Methods

### 2.1. Experimental conditions

This study builds upon the experimental framework and conditions established in the previous study by Kim and Yang [35]. Two distinct tip-in acceleration scenarios were employed, each characterized by a specific target acceleration level. The first experiment used a light tip-in condition, with a target acceleration of 0.1g. This scenario was designed to evaluate driver preferences under relatively low acceleration demands. The second experiment utilized a middle tip-in condition, with a target acceleration of 0.15g, simulating scenarios that require moderate acceleration and responsiveness. Typically, light tip-in corresponds to an accelerator pedal input of less than 30%, whereas middle tip-in corresponds to a pedal input range of 30% to 60%.

### 2.2. Acceleration profiles

For both experimental conditions, five longitudinal acceleration profiles from the previous study were adopted. These profiles were selected to represent a practical range suitable for real-world vehicle implementation, offering a robust basis for evaluating driver preferences:

- Profile A represents an ideal acceleration trajectory, designed to reach the target acceleration as rapidly as possible while emphasizing high responsiveness.
- Profile B serves as a benchmark for torque filter calibration, characterized by rapid initial acceleration followed by a smooth transition to the target acceleration.
- Profile C is designed to mitigate shocks induced by drivetrain backlash during the initial acceleration phase. It starts with a gradual initial acceleration to suppress backlash effects and then transitions smoothly to the target acceleration.
- Profile D follows a linear acceleration trajectory toward the target acceleration, reaching it within a time frame comparable to Profiles B and C.
- Profile E shares similarities with Profile B but integrates the initial acceleration level of Profile D with a more gradual overall acceleration trajectory, thereby delivering a smoother driving experience.

**Fig 1:**
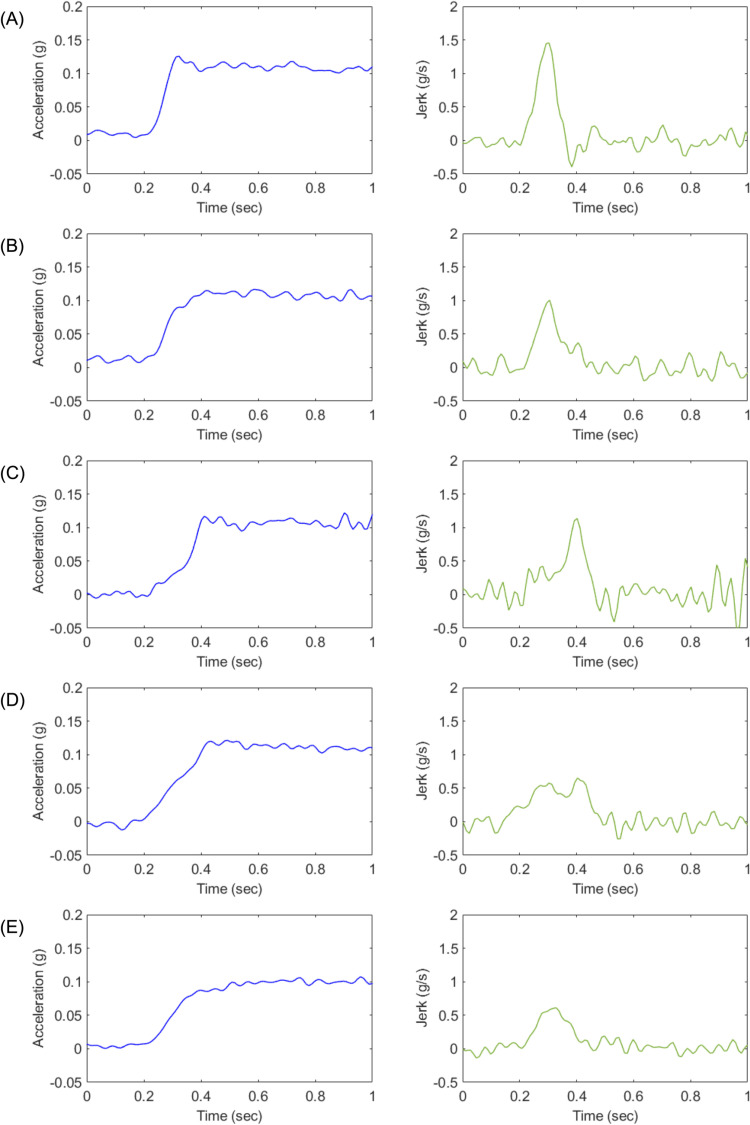
Longitudinal acceleration characteristics of each designed profile (A–E) for Experiment 1 [35].

**Fig 2:**
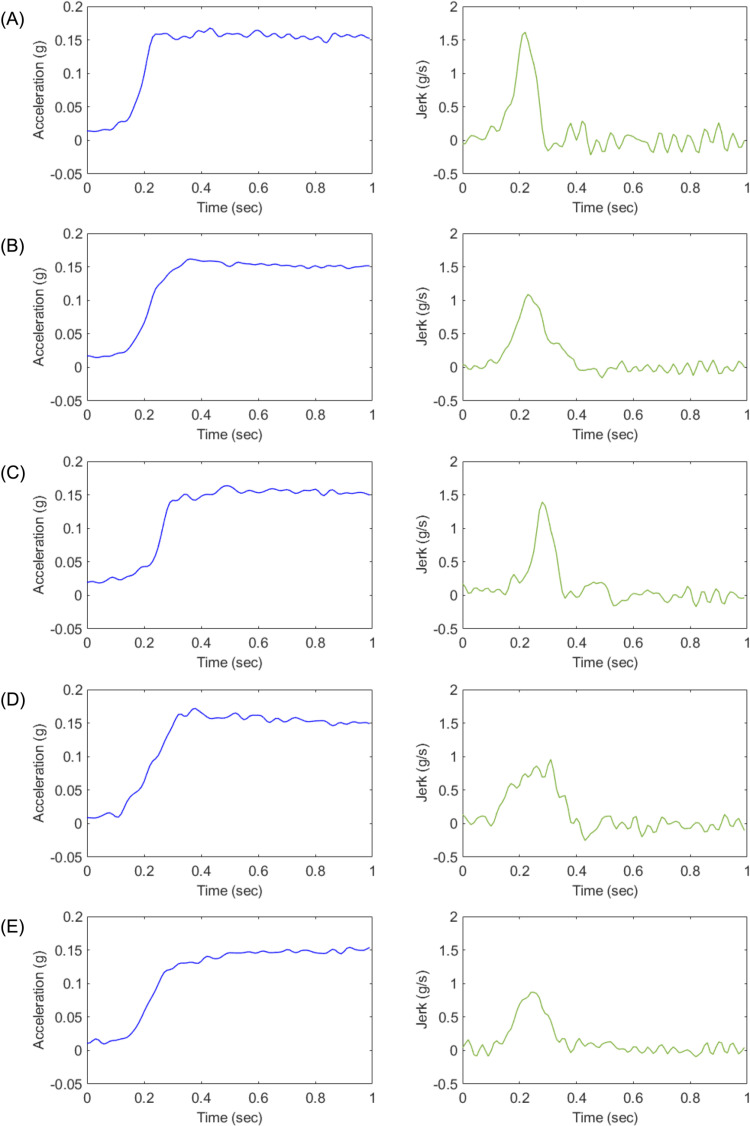
Longitudinal acceleration characteristics of each designed profile (A–E) for Experiment 2 [35].

Figs 1 and 2 illustrate the five acceleration profiles used in Experiment 1 and Experiment 2, respectively [35].

### 2.3. Subjects

The subjective evaluation included 15 experts with extensive experience in drivability development and assessment. To explore the influence of familiarity with specific vehicle types, the participants were divided into two groups based on their professional expertise. One group consisted of individuals primarily involved in the development of EVs, while the other group included individuals specializing in the development of ICEVs. All participants were male and had over a decade of professional experience in drivability development. They were well-versed in assessing longitudinal acceleration changes, ensuring consistency and reliability in their evaluations. Table 2 summarizes the demographic and professional backgrounds of the participants.

**Table 2:** Demographic and professional backgrounds of the participants.

Group	Number of subjects	Age range (years)	Median age (years)	Experience range (years)	Median experience (years)
**ICEV experts**	7	31-55	41	10-19	15
**EV experts**	8	31-40	37	10-15	12
**Total**	15	31-55	37	10-19	12

Participants were recruited voluntarily in response to an advertisement, and the experimental phase took place between April 1 and April 30, 2022. Before the evaluation, participants received detailed information about the purpose and procedures of the study, ensuring a clear understanding of their involvement. Written consent was obtained from all participants in accordance with the Declaration of Helsinki. The study was approved by the Institutional Review Board of Hyundai Motor Company and conducted in compliance with the company’s internal research protocol (HMC-IATD-2022-03-0002) [35].

### 2.4. Experimental procedure

**Fig 3:**
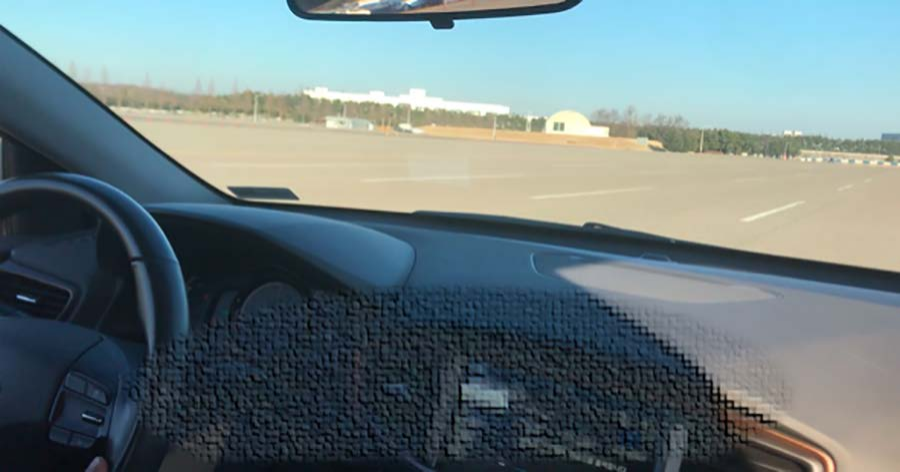
Interior view of the test vehicle during the evaluation of acceleration profiles.

The subjective evaluation was conducted at the proving ground of the Hyundai R&D Center in South Korea, utilizing real-world vehicle operations. Fig 3 illustrates the test environment used in this study. A hybrid electric vehicle (Hyundai Elantra Hybrid, 22MY, South Korea) was employed for the experiments, which were conducted exclusively in electric vehicle mode to assess the performance of the acceleration profiles. Using the paired comparison method, 10 evaluation pairs were systematically generated based on the formula N(N−1)/2, where N is the number of distinct acceleration profiles (*N* = 5). To minimize any bias arising from learning effects or fatigue, the sequence in which participants evaluated each pair was randomized. In Experiment 1, participants performed a light tip-in acceleration maneuver for approximately 5 seconds using the first acceleration profile from a designated pair, while maintaining a constant speed of 30 km/h. After reaching the target acceleration, participants decelerated, recorded their evaluations, and resumed driving at the same speed. The process was then repeated with the second acceleration profile of the pair, after which participants selected their preferred profile. This procedure was repeated for all evaluation pairs. Upon completing Experiment 1, participants took a brief rest before proceeding to Experiment 2, which followed the same evaluation process but under different experimental conditions.

## 3. Experimental results

The preference data from the paired comparison experiments were analyzed using the Bradley-Terry model to assess the relative preference strengths of the acceleration profiles for each evaluator group (EV experts and ICEV experts) [45]. The Bradley–Terry model represents each item with a latent parameter, commonly interpreted as its ability, and defines the probability of one item being preferred over another as a logistic function of the difference between their latent parameters. This model enabled the quantification of preference probabilities for the five profiles across the two experimental conditions [46,47]. In its basic formulation, the model assumes that there are n items, indexed by 1, 2, …, n. Each item i is characterized by a real-valued parameter $\theta_{i}$, which can be interpreted as a measure of its latent worth. When items i and j are presented side by side, the probability that i is chosen over j is specified by


$$P\left(Y_{ij}=1\right)=\frac{e^{\theta_{i}}}{e^{\theta_{i}}+e^{\theta_{j}}}$$ (1)

where $Y_{ij}=1$ denotes that item i is preferred over item j. By defining the probability in this manner, the model constrains all preference probabilities to lie between zero and one and ensures that the difference $\theta_{i}-\theta_{j}$ controls the log-odds of i beating j. In other words, a positive difference implies i is more likely to win, whereas a negative difference implies j is more likely to win. When $\theta_{i}$ and $\theta_{j}$ are equal, each item has a 50 percent chance of being chosen. A core feature of the Bradley–Terry model is the requirement of a constraint to achieve identifiability. Since only differences in parameters affect observed probabilities, it is not possible to estimate absolute parameter levels without further specification. Common approaches include fixing one parameter to zero or enforcing a constraint such as the sum-to-zero constraint


$$\sum_{i=1}^{n}\theta_{i}=0$$ (2)

These choices do not affect the model’s relative comparisons but merely establish a reference point or baseline.

Estimation of the item parameters is generally carried out through maximum likelihood methods. Suppose there are m observed pairwise comparisons. For each comparison k∈{1,…,m}, items $i_{k}$ and $j_{k}$ compete, and the binary outcome $y_{k}$ is 1 if $i_{k}$ is chosen, and 0 otherwise. The likelihood of the parameters $\theta =\left(\theta_{1},\theta_{2},\ldots ,\theta_{n}\right)$ can be written as


$$L\left(\theta \right)=\prod_{k=1}^{m}{\left(\frac{e^{{\theta_{i}}_{k}}}{e^{{\theta_{i}}_{k}}+e^{{\theta_{j}}_{k}}}\right)}^{y_{k}}{\left(\frac{e^{{\theta_{j}}_{k}}}{e^{{\theta_{i}}_{k}}+e^{{\theta_{j}}_{k}}}\right)}^{{1-y}_{k}}$$ (3)

Maximizing the log of this likelihood, subject to the chosen identifiability constraint, yields the maximum likelihood estimates of the $\theta_{i}$.

### 3.1. Experiment 1

Tables 3 and 4 present the analysis results for each evaluator group. To enhance visualization, centered estimates were calculated to improve interpretability. These estimates, obtained by enforcing the constraint $\sum_{i}\theta_{i}=0$, clarify which items lie above or below the average latent worth.

**Table 3:** Bradley-Terry model estimates for the group of ICEV experts in Experiment 1.

Profile	Centered Estimate (θ)	Standard Error	95% CI Lower	95% CI Upper	Order
**A**	−0.616240	0	−0.616240	−0.616240	5
**B**	−0.122484	0.505387	−1.113044	0.868075	4
**C**	−0.002852	0.504709	−0.992081	0.986378	2.5
**D**	0.744428	0.519705	−0.274194	1.763051	1
**E**	−0.002852	0.502946	−0.988626	0.982922	2.5

**Table 4:** Bradley-Terry model estimates for the group of EV experts in Experiment 1.

Profile	Centered Estimate (θ)	Standard Error	95% CI Lower	95% CI Upper	Order
**A**	−0.416129	0	−0.416129	−0.416129	5
**B**	0.312252	0.444292	−0.558560	1.183063	1.5
**C**	0.207753	0.442425	−0.659400	1.074907	3
**D**	0.312252	0.424669	−0.520100	1.144603	1.5
**E**	−0.416128	0.463082	−1.323768	0.491513	4

The results of the Bradley-Terry model analysis for the group of ICEV experts, as shown in Table 3, indicate distinct preferences for the acceleration profiles in Experiment 1. Profile D received the highest centered estimate (*θ* = 0.744), indicating a strong preference for this profile, which features a linear acceleration curve providing a balance of responsiveness and control. Profiles C and E shared the second-highest ranking (*θ* = −0.003), suggesting that ICEV experts valued profiles with gradual transitions and minimal shock. Profile B, which combines rapid initial acceleration with smooth progression, was ranked fourth (*θ* = −0.122), while Profile A, designed for rapid responsiveness, was ranked lowest (*θ* = −0.616), reflecting limited preference for highly dynamic acceleration characteristics in this group.

**Fig 4:**
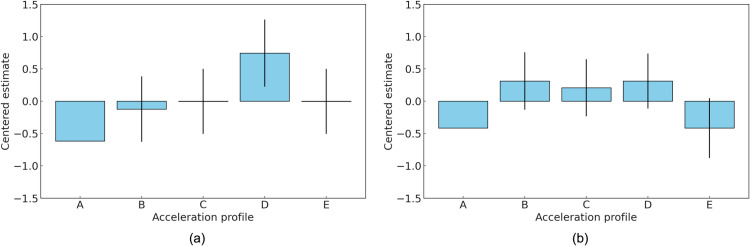
Centered estimates for the five acceleration profiles in Experiment 1. (a) Group of ICEV experts; (b) group of EV experts.

For the group of EV experts, as shown in Table 4, the preference distribution did not exhibit a clearly discernible pattern. Profiles B and D were equally ranked highest (*θ* = 0.312), indicating a shared inclination toward profiles that combine initial responsiveness with smooth transitions. Profile C, designed to mitigate shock during initial acceleration, was moderately preferred (*θ* = 0.208). Profiles A and E, which represent the extremes of rapid responsiveness and gradual acceleration, were the least preferred (*θ* = −0.416). The distribution of preferences among EV experts shows no strong inclination toward any specific profile. This observation might indicate that their evaluations are influenced by their familiarity with the broader performance characteristics typical of EV dynamics. Fig 4 illustrates the estimated values for the five acceleration profiles in Experiment 1, presented separately for each evaluator group.

**Fig 5:**
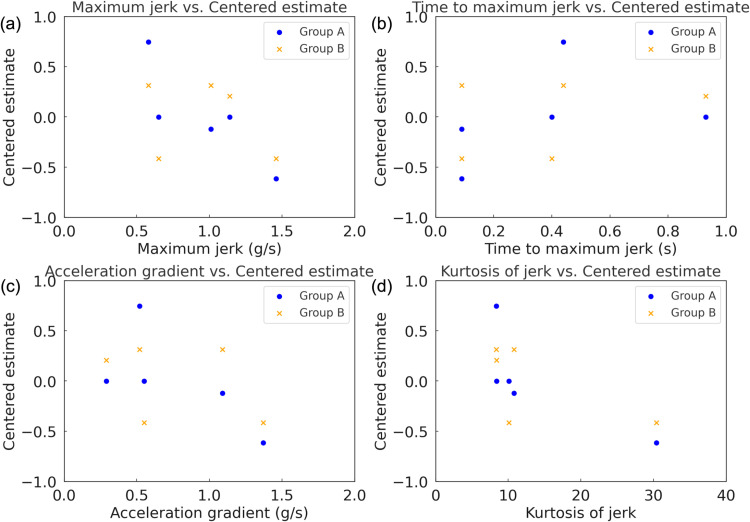
Scatter plots illustrating the relationships between dynamic characteristics of acceleration profiles and centered estimates of subjective preferences in Experiment 1. Subfigures show: (a) maximum jerk; (b) time to maximum jerk; (c) acceleration gradient; (d) kurtosis of jerk. Data points are distinguished by ICEV experts (Group A) and EV experts (Group B).

Following the preference analysis, the relationship between dynamic characteristics and profile preferences was evaluated. The analysis of dynamic characteristics in this study included maximum jerk, time to maximum jerk, acceleration gradient, and kurtosis of jerk. Maximum jerk represents the highest rate of change in acceleration during the evaluation period. Time to maximum jerk refers to the time elapsed from the beginning of the acceleration event to the point where maximum jerk occurs. The acceleration gradient indicates the rate at which acceleration changes over time, reflecting the steepness of the acceleration curve. Finally, kurtosis of jerk provides a statistical measure of the peakedness or tail distribution of jerk values throughout the event [48]. Fig 5 depicts the relationship between the dynamic characteristics of acceleration profiles and the subjective preferences recorded in Experiment 1. For the group of ICEV experts, the correlation analysis revealed significant insights into the factors influencing their preferences. A strong negative correlation (*r* = −0.8466) was observed between the maximum jerk and the centered estimate, indicating that profiles with higher maximum jerk were less preferred by this group. This finding suggests that ICEV experts tend to avoid acceleration profiles associated with abrupt changes in jerk. Similarly, the correlation between acceleration slope and centered estimate was also negative (*r* = −0.6775), highlighting a preference for profiles with gentler acceleration slopes. Furthermore, a strong negative correlation (*r* = −0.7543) was identified between jerk kurtosis and centered estimate, demonstrating that profiles offering smoother and more consistent jerk were favored. Specifically, the group of ICEV experts exhibited a strong preference for Profile D, which features a relatively smooth acceleration curve with moderate jerk levels and gradual acceleration. This profile closely resembles the traditional acceleration patterns of internal combustion engine vehicles, making it highly favorable among ICEV experts. Profile B also received relatively high preference scores from this group due to its smooth yet sufficiently responsive acceleration characteristics. These results highlight the ICEV experts’ preference for profiles that emphasize stability, smoothness, and predictability, which are attributes consistent with the conventional driving dynamics of ICEVs. Meanwhile, the expectations of the EV expert group regarding acceleration characteristics differed from those of the ICEV experts, reflecting the distinct dynamics of electric vehicles. The correlation analysis revealed differences in the relationship between dynamic characteristics and centered estimates. The correlation between maximum jerk and centered estimates for the EV experts was −0.2649, indicating a weaker negative association compared to the ICEV experts. This suggests that EV experts had a broader tolerance for profiles with higher levels of jerk. Likewise, the correlation between acceleration gradient and centered estimates was −0.3448, reflecting a continued, though less pronounced, aversion to steeper acceleration slopes compared to ICEV experts. The correlation between jerk kurtosis and centered estimates was –0.6318, indicating that although profiles with consistent and gradual changes were generally preferred, EV experts exhibited greater tolerance for dynamic variability. The EV experts demonstrated a clear preference for Profiles B and D. Profile B, in particular, received high ratings for its combination of rapid initial acceleration and stable progression, which are attributes that align well with the expected drivability characteristics of electric vehicles. The distinct characteristics of electric vehicles, particularly the emphasis on strong initial response and consistent power delivery, likely contributed to the group’s higher preference for Profiles B and D.

### 3.2. Experiment 2

**Fig 6:**
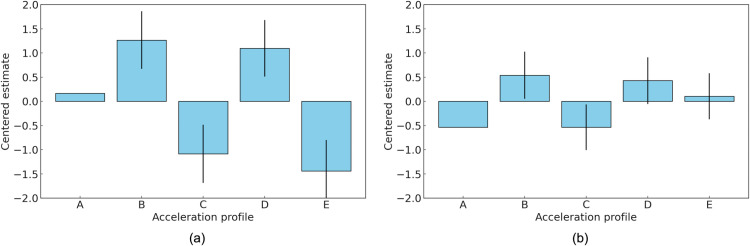
Centered estimates for the five acceleration profiles in Experiment 2. (a) Group of ICEV experts; (b) group of EV experts.

Subsequently, the results of Experiment 2 also indicated differences in the preference patterns between ICEV and EV experts under the middle tip-in condition, similar to the findings in Experiment 1. Tables 5 and 6 present the Bradley–Terry model estimates for the groups of ICEV experts and EV experts, respectively, in Experiment 2. The preference distribution for the five acceleration profiles showed that Profiles B and D were ranked higher in both groups, indicating a shared appreciation for profiles that balance responsiveness and stability under moderate acceleration demands. Profile B, characterized by its smooth progression and moderate acceleration dynamics, received the highest scores in both groups. Profile D, featuring a linear acceleration curve, was the second most preferred profile. In contrast, Profiles C and E, which emphasize gradual transitions and low jerk, were less favored overall. The ICEV experts exhibited strong preferences for Profiles B (*θ* = 1.265) and D (*θ* = 1.096), which align with their emphasis on smooth transitions and predictable dynamics. Profile B’s smooth yet dynamic progression is consistent with traditional ICEV acceleration expectations. Conversely, Profiles C (*θ* = −1.084) and E (*θ* = −1.442) were ranked the lowest, reflecting the group’s aversion to excessively gradual acceleration characteristics. In comparison, the EV experts also favored Profiles B (*θ* = 0.538) and D (*θ* = 0.428), but unlike the ICEV experts, they showed a slightly higher preference for Profile E (*θ* = 0.107), which features a linear and gradual acceleration curve. Profiles A and C, representing extremes in responsiveness and gradual transitions, were equally ranked lowest (*θ* = −0.536). This broader evaluation pattern among EV experts suggests a greater acceptance of profiles with varying characteristics. Fig 6 shows the estimated values for the five acceleration profiles in Experiment 2, presented separately for each evaluator group.

**Table 5:** Bradley-Terry model estimates for the group of ICEV experts in Experiment 2.

Profile	Centered Estimate (θ)	Standard Error	95% CI Lower	95% CI Upper	Order
**A**	0.165301	0	0.165301	0.165301	3
**B**	1.265184	0.59504	0.098906	2.431463	1
**C**	−1.084156	0.601887	−2.263855	0.095543	4
**D**	1.095616	0.583114	−0.047287	2.238520	2
**E**	−1.441946	0.641889	−2.700049	−0.183844	5

**Table 6:** Bradley-Terry model estimates for the group of EV experts in Experiment 2.

Profile	Centered Estimate (θ)	Standard Error	95% CI Lower	95% CI Upper	Order
**A**	−0.536496	0	−0.536496	−0.536496	4.5
**B**	0.538461	0.486448	−0.414976	1.491899	1
**C**	−0.536496	0.473004	−1.463583	0.390591	4.5
**D**	0.427879	0.48281	−0.518427	1.374186	2
**E**	0.106651	0.47606	−0.826427	1.039729	3

**Fig 7:**
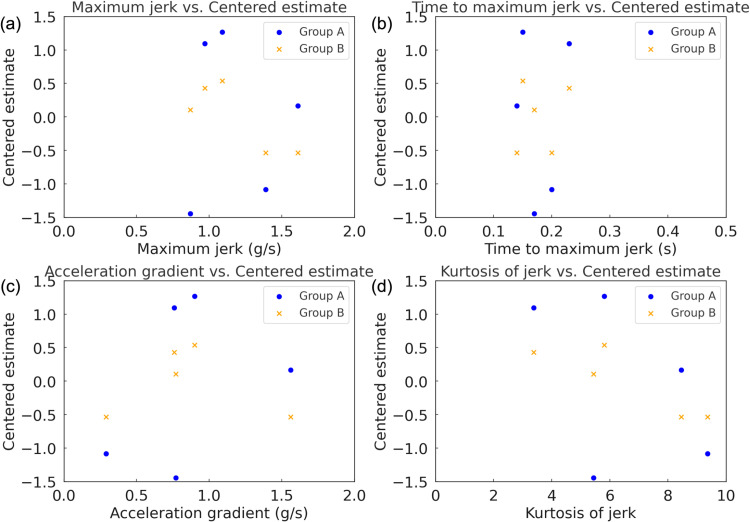
Scatter plots illustrating the relationships between dynamic characteristics of acceleration profiles and centered estimates of subjective preferences in Experiment 2. Subfigures show: (a) maximum jerk; (b) time to maximum jerk; (c) acceleration gradient; (d) kurtosis of jerk. Data points are distinguished by ICEV experts (Group A) and EV experts (Group B).

Furthermore, the correlation analysis for the group of ICEV experts in Experiment 2 indicated only weak relationships between certain dynamic characteristics and centered estimates. Fig 7 illustrates the correlation between dynamic characteristics of acceleration profiles and subjective preferences observed in Experiment 2. The correlation coefficient between maximum jerk and centered estimates was −0.038, indicating almost no relationship. In contrast, the acceleration gradient showed a positive correlation of 0.356, suggesting that this group exhibited a relative preference for profiles with steeper acceleration slopes. Additionally, the correlation coefficient between jerk kurtosis and centered estimates was −0.465, indicating that profiles with fewer abrupt changes and smoother transitions were generally preferred by the ICEV experts. For the EV experts group, the correlation analysis indicated stronger and more distinct relationships between dynamic characteristics and centered estimates. A strong negative correlation (*r* = −0.816) was observed between maximum jerk and centered estimates, suggesting that the EV experts strongly disfavored profiles with abrupt changes in jerk. The correlation coefficient between time to maximum jerk and centered estimates was 0.196, indicating a slight tendency for profiles with longer times to reach maximum jerk to be more preferred. Lastly, the correlation coefficient between jerk kurtosis and centered estimates was −0.886, reflecting a clear preference for profiles with smooth and consistent jerk characteristics among the EV experts.

These results indicate notable differences between the two groups in their evaluation of dynamic characteristics. The ICEV experts exhibited tolerance for steeper acceleration gradients, likely reflecting their familiarity with traditional ICEV dynamics. On the other hand, the EV experts demonstrated strong aversions to abrupt changes in jerk and a clear preference for smoother and more consistent acceleration profiles, aligning with the inherent characteristics of EV drivability. These findings align with the inherent characteristics of EV drivability, which is characterized by smooth power delivery due to the absence of gear transitions and the use of electric motors that provide immediate and linear torque. The strong aversions to abrupt changes in jerk and the preference for smoother profiles observed among EV experts likely stem from their familiarity with these distinct dynamics. This suggests that the EV experts’ evaluation patterns are shaped by their experiences with the predictable and consistent performance typically associated with electric vehicles.

## 4. Discussion

### 4.1. Implications of the results

**Fig 8:**
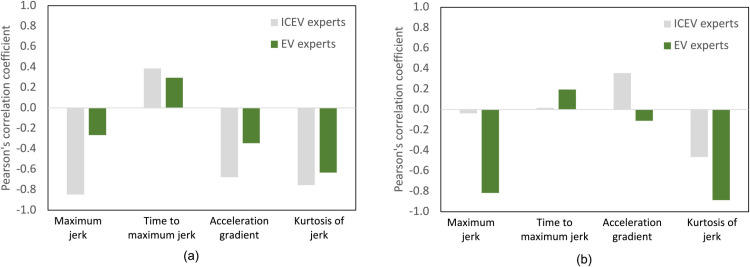
Pearson correlation coefficients for dynamic characteristics of acceleration profiles in Experiments 1 and 2. (a) ICEV and EV expert groups under the light tip-in condition (Experiment 1); (b) ICEV and EV expert groups under the middle tip-in condition (Experiment 2). Dynamic characteristics include maximum jerk, time to maximum jerk, acceleration gradient, and jerk kurtosis.

The findings from Experiments 1 and 2 highlight distinct differences in the preferences and evaluations of dynamic characteristics between ICEV and EV experts. These differences are further clarified by the Pearson’s correlation coefficients for dynamic characteristics, as summarized in Fig 8. Subfigure (a) corresponds to Experiment 1, while (b) represents Experiment 2. In Experiment 1, ICEV experts showed a clear preference for Profile D, reflecting their emphasis on stability and predictability in acceleration. In contrast, EV experts favored Profiles B and D, which align with their preference for a combination of responsiveness and smoothness, consistent with EV drivability. Both groups ranked Profile A, characterized by rapid initial responsiveness, as the least preferred, though ICEV experts exhibited a stronger dislike for this profile. Moreover, experiment 2 showed similar patterns but with notable nuances. ICEV experts preferred Profiles B and D, which emphasize smooth progression and balanced acceleration, while showing a lower preference for Profiles C and E due to their gradual transitions, which did not align with traditional ICEV dynamics. EV experts also preferred Profiles B and D, but they showed a slightly higher preference for Profile E, which features a linear and gradual acceleration curve. Both groups continued to rank Profiles A and C lowest, indicating a shared lack of favor for extremes in either rapid responsiveness or overly gradual transitions. The correlation analysis provided further insights into these preferences. In Experiment 1, ICEV experts displayed a strong negative correlation between maximum jerk and centered estimates, indicating their dislike for profiles with sudden changes in jerk. The moderate negative correlation with the acceleration gradient reinforced their preference for smoother transitions. In contrast, EV experts showed weaker correlations with maximum jerk and acceleration gradient, suggesting a broader tolerance for these dynamic characteristics. However, both groups exhibited a strong negative correlation with jerk kurtosis, indicating a shared preference for profiles that minimize sudden variability and provide consistent acceleration dynamics. In Experiment 2, ICEV experts showed negligible correlation between maximum jerk and centered estimates, reflecting minimal sensitivity to sudden changes at moderate acceleration levels. A positive correlation with acceleration gradient suggested a preference for steeper acceleration slopes, likely reflecting traditional ICEV performance characteristics. EV experts, however, demonstrated a strong negative correlation with maximum jerk, highlighting their low preference for sudden acceleration changes. The continued strong negative correlation with jerk kurtosis emphasized their preference for smooth and consistent jerk distributions. Additionally, a slight positive correlation with time to maximum jerk suggested that gradual transitions were marginally favored. To support these observations, Table 7 presents the Pearson correlation coefficients between subjective preference scores and four key dynamic parameters: maximum jerk, time to maximum jerk, acceleration gradient, and jerk kurtosis, categorized by group and tip-in acceleration condition. Under the light tip-in condition, ICEV experts exhibited strong negative correlations with both maximum jerk (*r* = −0.85) and jerk kurtosis (*r* = −0.75), suggesting heightened sensitivity to abrupt transients in vehicle behavior. EV experts under the same condition showed weaker correlations ((*r* = −0.85) and *r* = −0.63 respectively), indicating relatively greater tolerance for dynamic variability. In contrast, under the middle tip-in condition, EV experts displayed strong negative correlations with both maximum jerk (*r* = −0.82) and jerk kurtosis (*r* = −0.89), whereas ICEV experts demonstrated minimal associations, with correlations close to zero. These results provide quantitative evidence for the divergence in perceptual sensitivity and preference formation between the two groups.

**Table 7:** Pearson correlation coefficients between centered estimates and dynamic profile characteristics.

Group	Acceleration Condition	Max. jerk (*r*)	Time to max. jerk (*r*)	Acceleration gradient (*r*)	Jerk kurtosis (*r*)
**ICEV Experts**	Light Tip-in Acceleration	–0.85	0.39	–0.68	–0.75
**EV Experts**	Light Tip-in Acceleration	–0.26	0.30	–0.34	–0.63
**ICEV Experts**	Middle Tip-in Acceleration	–0.04	0.02	0.36	–0.46
**EV Experts**	Middle Tip-in Acceleration	–0.82	0.20	–0.11	–0.89

The findings from this study, which indicate that EV experts show lower preferences for rapid acceleration and high jerk, align with existing research emphasizing the importance of comfort and smooth transitions in EV drivability. While EVs are capable of delivering high torque instantly, studies have shown that such rapid acceleration can lead to excessive jerk, negatively impacting passenger comfort and control. For example, Batra highlighted the need for anti-jerk control systems to improve drivability by minimizing torsional oscillations caused by abrupt torque changes [49]. Similarly, Chakraborty et al. demonstrated that smooth acceleration strategies, which balance energy efficiency and driving comfort, are often preferred over aggressive acceleration profiles [50]. These findings reinforce the idea that experienced EV users value drivability characteristics such as smooth power delivery and gradual transitions, emphasizing a balance between performance and comfort to meet broader user expectations.

In this context, the findings of this study further highlight the influence of developmental background and vehicle familiarity on acceleration profile preferences. ICEV experts consistently prioritized vehicle stability and control, favoring profiles with predictable dynamics under low acceleration conditions and steeper slopes under moderate demands. In contrast, EV experts demonstrated preferences aligned with EV drivability, favoring profiles that balance responsiveness with smoothness while avoiding abrupt changes. Together, these results underscore the importance of designing acceleration profiles that account for diverse user preferences, particularly in the transition between ICEV and EV technologies. Building on these findings, incorporating dynamic characteristics that align with the preferences of both ICEV and EV experts can contribute to the development of EV designs that address the expectations of a broader range of users. By achieving a balance between performance, comfort, and adaptability, such designs have the potential to support a smoother transition for users adopting EV technologies, thereby enhancing overall user satisfaction and acceptance.

### 4.2. Limitations and future works

This study has several limitations that should be taken into consideration. One notable limitation is the small sample size of 15 evaluators, which, while providing expert insights, may not fully represent the broader spectrum of consumer preferences. Expanding the participant pool to include a larger and more diverse range of individuals, particularly general consumers, could offer a more comprehensive understanding of preferences. Another limitation lies in the reliance on subjective evaluations, which can be influenced by individual biases or perceptions. To enhance the reliability of findings, future studies could integrate physiological measures, such as heart rate or stress levels, to complement subjective assessments and provide a more objective evaluation. Furthermore, this study was conducted in a controlled experimental setting, which may not fully capture the complexities of real-world driving scenarios. Evaluating acceleration profiles in actual driving environments, encompassing diverse road conditions and traffic situations, would yield findings that are more reflective of practical applications. The study’s focus on a specific set of acceleration profiles provided valuable insights but does not encompass the entire range of vehicle dynamics or user requirements. Including additional factors such as regenerative braking, speed variability, and other dynamic characteristics in future studies would deepen the understanding of EV drivability and its impact on user preferences. Finally, the study was limited to EV and ICEV experts and did not account for potential cultural or regional influences on acceleration preferences. Expanding research to include participants from diverse cultural and geographic backgrounds could provide a more global perspective on consumer preferences. Addressing these limitations in future research will deepen the understanding of acceleration profile preferences and facilitate the development of electric vehicle designs that are aligned with diverse user needs and expectations.

## 5. Conclusion

This study examined acceleration profile preferences among ICEV and EV experts, shedding light on how developmental background and vehicle familiarity influence evaluations of dynamic characteristics. Through subjective assessments conducted under two experimental conditions, notable differences were identified between the two groups. ICEV experts consistently prioritized stability, predictability, and control, favoring profiles with smooth transitions under low acceleration conditions and steeper slopes under moderate demands. In contrast, EV experts preferred profiles that balance responsiveness with smoothness, aligning with the characteristics of EV drivability, which emphasize gradual transitions and consistent power delivery. Both groups shared a mutual dislike for extremes in either rapid responsiveness or overly gradual transitions, highlighting the importance of balanced designs. Correlation analyses further supported these observations. Under the light tip-in condition, ICEV experts showed strong negative correlations between preference and both maximum jerk (*r* = −0.85) and jerk kurtosis (*r* = −0.75), indicating heightened sensitivity to abrupt changes. EV experts, by contrast, showed weaker correlations (*r* = −0.26 and *r* = −0.63), suggesting a more tolerant and adaptive approach to drivability cues. Under the middle tip-in condition, EV experts demonstrated strong negative correlations with both maximum jerk (*r* = −0.82) and jerk kurtosis (*r* = −0.89) whereas ICEV experts exhibited minimal correlations, indicating a shift in sensitivity depending on the acceleration context. These results challenge the assumption that EV users universally favor rapid acceleration, instead pointing to nuanced preferences that prioritize comfort and control. The findings of this study have meaningful implications for the development of user-focused EV designs. By incorporating dynamic characteristics that accommodate the preferences of both ICEV and EV experts, acceleration profiles can be adjusted to better address diverse user expectations and encourage the adoption of EV technologies. Future studies should expand on these findings by involving larger and more diverse participant groups, exploring real-world driving conditions, and integrating additional dynamic factors such as regenerative braking and speed variability. These efforts will enhance the understanding of acceleration preferences and contribute to the design of EVs that meet a broader range of user needs.
